# cGMP and cAMP pathways rearrange ARHGAP17 and ARHGEF6 protein complexes to control Rac1 in platelets

**DOI:** 10.1186/2050-6511-16-S1-A16

**Published:** 2015-09-02

**Authors:** Zoltan Nagy, Kieran Wynne, Alexander von Kriegsheim, Stepan Gambaryan, Albert Smolenski

**Affiliations:** 1 School of Medicine, University College Dublin, Dublin, Ireland; 2 Conway Institute, University College Dublin, Dublin, Ireland; 3 Systems Biology Ireland, University College Dublin, Dublin, Ireland; 4 Sechenov Institute of Evolutionary Physiology and Biochemistry, Russian Academy of Sciences, St. Petersburg, 194223, Russia

## 

Endothelial cells inhibit blood platelets by releasing nitric oxide (NO) and prostacyclin (PGI2). NO and PGI2 activate cGMP and cAMP synthesis, leading to the activation of cGMP- and cAMP-dependent protein kinases (PKG, PKA), phosphorylation of substrate proteins, and inhibition and reversal of platelet activation and aggregation. A known target of PKA and PKG is the small G-protein Rac1. The active form of Rac1, Rac1-GTP, contributes to cytoskeletal reorganization, platelet spreading, adhesion, granule release, and aggregation. PKG and PKA activation have been shown to reduce Rac1-GTP levels. Rac1-GTP is controlled by GTPase-activating proteins (GAPs - reduce Rac1-GTP) and Guanine-nucleotide exchange factors (GEFs - elevate Rac1-GTP). We hypothesized that specific GAPs and/or GEFs might mediate the inhibitory effects of PKG and PKA on Rac1. Screening for potential substrates among the GAPs and GEFs expressed in platelets resulted in the identification of the GAP ARHGAP17 (also known as Rich1 or Nadrin) as well as the GEF ARHGEF6 (also known as alpha-PIX or Cool2) as new substrates of PKG and PKA in human platelets. We mapped the phosphorylation sites on ARHGAP17 and ARHGEF6, and we investigated the functional consequences of phosphorylation. Phosphorylation of ARHGAP17 by PKA resulted in a dissociation of ARHGAP17 and its interaction partner CIP4. Phosphorylation of ARHGEF6 by both kinases resulted in the association of the ARHGEF6/GIT1 complex with the phospho-serine/threonine binding protein 14-3-3.

Our data suggest that PKG- and PKA-mediated phosphorylations of ARHGAP17 and ARHGEF6 lead to a reorganization of protein complexes involved in the control of Rac1. These events are likely to contribute to Rac1 inhibition by cyclic nucleotides. Similar findings regarding the regulation of a critical small G-protein by GAP and GEF phosphorylation have been described for Rap1 in platelets before. We conclude that phosphorylation of GAPs and GEFs of small G-proteins might represent a general principle of platelet regulation by cGMP and cAMP.

